# Impact of GLP-1 receptor agonists on patients with intraductal papillary mucinous neoplasms: Study protocol for a multicentric cohort study

**DOI:** 10.1371/journal.pone.0357577

**Published:** 2026-09-09

**Authors:** Melissa Lagger, Hugo Najberg, Almir Miftaroski, Lucien Widmer, Sara Di Renzo, Pouya Iranmanesh, Maxime Matter, Boran Tekdogan, Stéphanie Perrodin, Beat Gloor, Christian Toso, Jean-Louis Frossard, Michel Adamina, Leo Buhler

**Affiliations:** 1 Department of Surgery, Fribourg Cantonal Hospital, Villars-sur-Glâne, Switzerland; 2 Department of Medical and Surgical Specialties, Faculty of Science and Medicine, University of Fribourg, Fribourg, Switzerland; 3 Direction of IT service Education and Research, University of Fribourg, Fribourg, Switzerland; 4 Department of Radiology, Fribourg Cantonal Hospital, Villars-sur-Glâne, Switzerland; 5 Hirslanden Clinics Geneva, Geneva, Switzerland; 6 Universitätsklinik für Viszerale Chirurgie und Medizin, Universität Bern, Inselspital, Bern, Switzerland; 7 Division of Digestive Surgery, University Hospitals of Geneva, Genève, Switzerland; 8 Division of Gastroenterology, University Hospitals of Geneva, Genève, Switzerland; National Institutes of Health, UNITED STATES OF AMERICA

## Abstract

**Aim of the study:**

Intraductal papillary mucinous neoplasms (IPMNs) are among the most common cystic pancreatic neoplasms, with a potential to progress to malignancy. The increasing prevalence of IPMNs, coupled with the widespread use of GLP-1 receptor agonists (GLP-1 RAs) for diabetes and obesity management, raises concerns about the safety of these medications in patients with preexisting pancreatic conditions. Despite their proven metabolic benefits, questions remain about their impact on pancreatic pathology. This study investigates the association between GLP-1 RA use and IPMN progression to address this critical gap in the current literature.

**Methods:**

This retrospective multicentric cohort study will analyse data from January 2010 to July 2026 across three Swiss tertiary institutions. Patients with a radiological diagnosis of IPMN and/or treated with GLP-1 RAs will be included. Patients will be stratified into four groups according to a 2 × 2 design based on GLP-1 RAs exposure and IPMN status. The primary objective is to evaluate the impact of GLP-1 RAs on IPMN progression using radiological criteria from the Kyoto 2023 Consensus. Secondary objectives include assessing changes in tumour markers (CA19−9, CEA), the incidence of acute pancreatitis, the progression of IPMNs to high-grade dysplasia or invasive carcinoma, and the need for surgical intervention or altered surveillance protocols associated with GLP1 RAs use.

**Discussion:**

This study aims to explore potential associations between GLP-1 RA use and changes in IPMN characteristics, tumour markers, and disease progression. Given the retrospective design and expected small sample size (estimated at 30–60 patients in total), the study may not be powered to establish definitive correlations. Nonetheless, it will generate preliminary data to help inform hypotheses and guide future research. Any observed trends could provide valuable insights into the safety of GLP-1 RAs in patients with IPMNs and contribute to more informed clinical decision-making regarding the use of these agents in a population at risk for pancreatic disease progression.

**Trial registration:**

ClinicalTrials.gov NCT07014709

## Introduction

Intraductal papillary mucinous neoplasms (IPMNs) are among the most common cystic pancreatic neoplasms, notable for their potential to progress to malignancy, particularly in main-duct subtypes [1–3]. The incidence of IPMNs has risen significantly, partly due to advancements in imaging technology [4]. Chronic pancreatitis is recognized as a relevant risk factor for the development of IPMN, likely due to the inflammatory changes that alter the pancreatic ductal epithelium [5,6].

The widespread adoption of Glucagon-like peptide-1 receptor agonists (GLP-1 RAs) for diabetes since 2005 and more recently obesity management has raised questions about their safety in patients with preexisting pancreatic conditions. While GLP-1 RAs are effective in promoting weight loss and metabolic improvement, concerns about their potential links to acute pancreatitis (AP) and pancreatic cancer remain unresolved. Although recent meta-analyses [7,8] have not demonstrated a definitive association between GLP-1 RAs and acute pancreatitis, no study specifically addressed their safety in patients with preexisting IPMNs.

The relationship between GLP-1 RAs and pancreatic cancer remains uncertain. While recent meta-analyses [9,10] have not demonstrated a definitive association between GLP-1 RAs use and pancreatic cancer, pharmacovigilance data have suggested a potential signal, with an increased reporting rate of pancreatic malignant neoplasms in association with GLP-1 RAs exposure. The definitive mechanism underlying a potential association between GLP-1 RAs and pancreatic neoplasia has not been established. Proposed hypotheses include a possible link mediated through pancreatitis, a known risk factor for pancreatic cancer, as well as experimental data suggesting potential pro-inflammatory and proliferative effects within pancreatic tissue. However, these findings are inconsistent across studies, and no causal mechanism has been confirmed. [11,12]

MRI, along with physical examination, assessment of tumour marker and new onset diabetes are the preferred ways to provide surveillance for IPMN [1]. Shi X. [13] reported the case of a woman in her mid-60s with diabetes, treated with dulaglutide injections for 16 months, which presented with asymptomatic markedly elevated serum CA19−9 and CA242 levels revealed during a routine health examination. Imaging and interventional assessments did not reveal any hepatobiliary, gastrointestinal or pancreatic neoplasm. Discontinuation of dulaglutide, resulted in normalisation of serum CA19−9 and CA242 levels within 6 weeks. This elevation in a patient under surveillance for IPMN and treated with GLP-1 RAs could complicate the differentiation between benign condition and malignant transformation, making clinical interpretation challenging.

Animal studies have not clarified the understanding of GLP-1 RAs’ effects on the pancreas. Rodent models have shown inconsistent results, ranging from exacerbation of pancreatitis [14] and promotion of pancreatic duct gland growth [15,16] to anti-inflammatory effects that reduce chemically induced pancreatitis [17]. While nonhuman primate studies suggest minimal impact on pancreatic structure, they are limited in scope and do not fully replicate human physiology [18,19]. The potential for GLP-1 RAs to influence pancreatic ductal cells, as observed in animal studies, warrants cautious interpretation and further investigation in the context of IPMNs.

Given the increasing prevalence of both IPMNs and GLP-1 RA usage, and the complex interplay between inflammation, neoplastic progression, and potential medication effects, further investigation is critical. A retrospective study exploring outcomes in patients with IPMNs who have been treated with GLP-1 RAs for diabetes or obesity will provide much-needed insight into the safety profile of these medications in this unique and potentially vulnerable population.

## Methods

### Study design

This is a retrospective multicentric cohort study conducted at three Swiss Hospitals, Cantonal Hospital of Fribourg (HFR), University Hospital of Geneva (HUG) and University Hospital of Bern (Inselspital), to evaluate the effects of GLP-1 RA therapy on patients with IPMNs. Data from January 2010 to July 2026 will be reviewed.

The primary focus of the study will be to investigate the impact of GLP-1 RA therapy on the progression of IPMNs, specifically examining changes in IPMN radiological characteristics such as described in the Kyoto 2023 Consensus [1]. Additionally, the study will assess the variations in serum tumor markers such as CA19−9 and CEA and incidence of acute pancreatitis in patients undergoing GLP-1 RA treatment. The progression of IPMNs to high-grade dysplasia or invasive carcinoma, the need for surgical intervention or changes in IPMN surveillance protocols will also be assessed.

**Fig 1:**
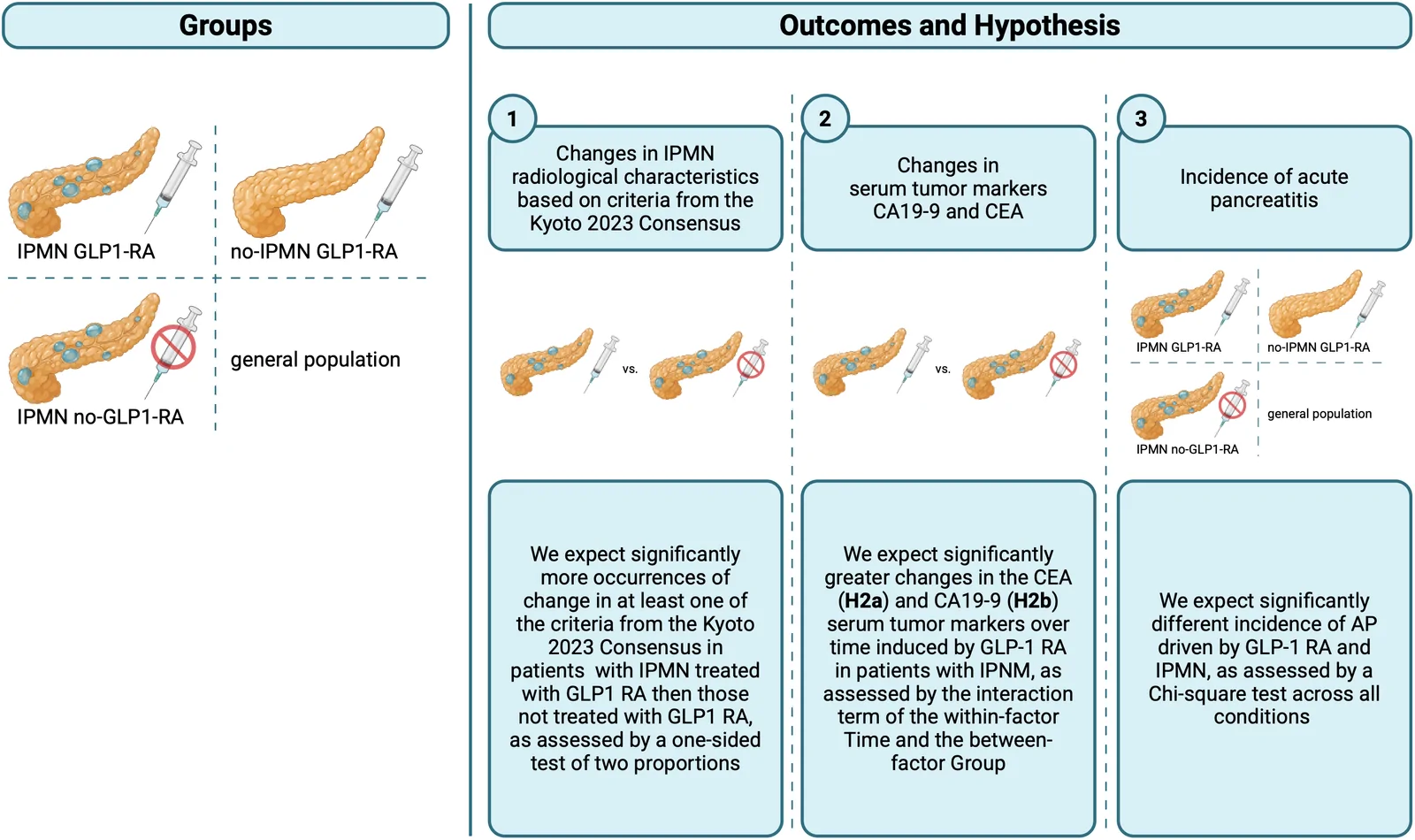
Study design and comparison groups. Schematic overview of the retrospective multicentric cohort study evaluating the impact of GLP-1 RA therapy on patients with IPMNs. Four study groups will be included according to IPMN diagnosis and GLP-1 RA exposure: patients with IPMN treated with GLP-1 RAs, patients with IPMN not treated with GLP-1 RAs, patients without IPMN treated with GLP-1 RAs, and patients without IPMN and without GLP-1 RA exposure (general population). The primary outcome (1) is the progression of radiological IPMN characteristics according to the Kyoto 2023 Consensus criteria. Secondary outcomes include longitudinal changes in serum CA19−9 and CEA levels (2) and the incidence of acute pancreatitis (3). We hypothesize that GLP-1 RA therapy is not associated with an increased progression of IPMN radiological features or adverse pancreatic outcomes compared with patients not exposed to GLP-1 RAs.

Data already collected from medical records, laboratory results, and imaging studies will be reviewed for patients diagnosed with IPMNs and treated with GLP-1 RAs for diabetes or obesity between 01.01.2010 and 31.07.2025 at HFR, HUG, and Inselspital. To assess the impact of GLP-1 RA exposure in patients with IPMNs, we will also include control groups without GLP-1 RA exposure and without IPMNs, resulting in four groups defined by a 2 × 2 factorial design (GLP-1 RA exposure: yes/no × IPMN: yes/no) (Fig 1).

A list of patients diagnosed with IPMNs will be compiled (List A), and this group will then be cross-referenced with data from patients who have been treated with GLP-1 RAs (List B). ICD-10 codes, keywords like IPMN and ATC medication codes will be used to identify the patients and compile the two lists (S1 File). This method will allow us to identify the subset of patients in whom both IPMN and GLP-1 RA treatment are present. The same number of patients will be randomly extracted from the two lists. For example, if 3 patients with GLP1-RA and IPMN are extracted in the HFR dataset, then 3 patients will be extracted in List A and 3 in List B. The same procedure will be carried out for the other two institutions. For the no GLP-1 RA & no IPMN group, the proportion of AP will be generalized to the incidence in the general population.

Evaluation methods will include a review of medical records for demographic data (age, sex, BMI), clinical and family history, and comorbidities (diabetes, dyslipedmia, hypertension), along with laboratory values (lipase, amylase, HbA1c, fasting glucose, HOMA-IR, plasma insulin, C-peptide, CEA, CA 19−9). Radiological data, including data from MRI scans, will be used to monitor changes in IPMN characteristics. If only CT scans are available, they will also be evaluated, even if this is not the preferred imaging method for IPMN evaluation. Gastroenterological examination including EUS and cytological results will also be reviewed. This study adheres to the SPIRIT 2013 recommendations.

### Population

Inclusion criteria are adult patients (≥18 years old) with a radiological diagnosis of IPMN and/or documented treatment with GLP-1 RAs, availability of medical records including laboratory results and imaging studies and signed informed consent permitting the use of anonymized clinical data for research. Eligible individuals will then be classified into one of four groups:

- Patients with GLP-1 RA exposure and IPMN
- Patients with GLP-1 RA exposure without IPMN
- Patients without GLP-1 RA exposure with IPMN
- Patients without GLP-1 RA exposure and without IPMN = general population

Exclusion criteria is refusal of consent for research participation.

### Data collection

Data will be extracted from electronic medical records and include demographic information (age, sex, BMI), clinical and family history, comorbidities (e.g., diabetes, dyslipidemia, hypertension), laboratory values (HbA1c, fasting glucose, lipase, amylase, CA19−9, CEA), and imaging findings. Study data will be collected and managed using REDCap electronic data capture tools hosted at University of Fribourg [20,21]. REDCap database is available as S2 File. Data collection will start on September 2025 and is expected to be completed by March 2026. Results are expected to be published by November 2026. S3 File illustrates a detailed schedule for enrolment, data collection and assessment.

### Outcomes

#### Primary outcome.

Changes in IPMN radiological characteristics based on criteria from the Kyoto 2023 Consensus [1].

#### Secondary outcomes.

1. Changes in serum tumor markers (CA19-9, CEA) over time
2. Incidence of AP

### Hypotesis

Our Hypothesis are illustrated in Fig 1.

### Radiological evaluation

MRI is the preferred imaging modality; CT scans will be included if MRI is unavailable. Two senior radiologists at HFR will independently and blinded to prior findings assess imaging studies with the internationally recognized semiological criteria for assessing cystic pancreatic lesions on cross-sectional imaging: high-risk stigmata and worrisome features (cyst diameter, enhancing mural nodules, thickened or enhancing cyst walls, main pancreatic duct diameter, change in calibre, lymphadenopathy, cystic growth). In addition, morphological and signal analysis criteria of the pancreatic gland will be analysed (glandular volume, T1 and T2 weighted signal, diffusion restriction). Furthermore, radiological assessments will be used to monitor for any signs of pancreatitis, such as pancreatic inflammation or fluid collections, that may be associated with GLP-1 RA therapy.

### Sample size and statistical analysis

#### Sampling plan.

The sample size is expected to be between 10 and 20 patients for the condition with GLP-1 RAs and IPMN, due to the rare nature of the patient population with both IPMN and GLP-1 RA use. The other conditions (GLP-1 RA & no-IPMN, no GLP-1 RA & IPMN) will match in sample size the above-mentioned condition as it is the most stringent. This design ensures to isolate the interaction effect of GLP-1 RA and IPMN while ensuring proper statistical power. Indeed, a-priori power analysis computed with G*Power [22] shows that for our smallest estimation of effect size (i.e., 10–30 patients per condition, 30–60 in total), a 2x2 ANOVA detects a significant medium effect size with a power if 80% and an alpha of 0.05.

#### Positive controls.

Groups need to balanced in age, BMI, and in their number of follow-ups. If age between groups is unbalanced, as defined by a Cohen’s d > 0.4, outliers will be removed until this threshold is reached. For the number of follow-ups, follow-ups above the limiting factor will be ignored from the data collection to balance the groups if needed.

#### Analysis plan.

H1) We expect significantly more occurrences of change in at least one of the criteria from the Kyoto 2023 Consensus (see Outcomes section) [1] from patients with GLP-1 RA & IPMN than patients with no GLP-1 RA & IPMN, as assessed by a one-sided test of two proportions. H2) We expect significantly greater changes in the CEA (H2a) and CA19−9 (H2b) serum tumor markers over time induced by GLP-1 RA, as assessed by the interaction term of the within-factor Time and the between-factor Group (GLP-1 RA & IPMN vs. no GLP-1 RA & IPMN). H3) We expect significantly different incidence of AP driven by GLP-1 RA and IPMN, as assessed by a Chi-square test across all conditions (GLP-1 RA & IPMN, no GLP-1 RA & IPMN, GLP-1 RA & no IPMN).

Timing of progression: T0 will be defined as the first available follow-up examination for patients with IPMN who were not treated with GLP-1 RAs, and as the first available follow-up examination after initiation of GLP-1 RA therapy for patients with IPMN who received GLP-1 RAs.

### Ethics approval and consent to participate

The Ethic Committee “Commission cantonale d’éthique de la recherche sur l’être humain (CER-VD)” has approved this retrospective study (Application number 2025−00486) and given consent to participate. All patients have signed the general consent from one of the three participating institutions (S4 File). All modifications to the protocol will be communicated to the ethics committee as amendments. Data transfer agreement was signed by the three primary investigators at the three institutions prior to the beginning of the study. All data are handled with study ID and without identification number.

## Discussion

This study aims to provide preliminary insights into the potential impact of GLP-1 RAs therapy on the progression and clinical outcomes of IPMNs. A better understanding of the relationship between GLP-1 RA exposure and IPMNs may help address current safety uncertainties and support clinical decision-making in this complex patient population.

Several limitations must be acknowledged. The retrospective design introduces inherent risks of selection and information bias, while the relatively small sample size limits statistical power and the ability to detect modest associations. Patient identification may be incomplete, as GLP-1 RAs prescriptions may have been initiated by healthcare providers outside the participating institutions or inconsistently documented and therefore not fully captured in institutional records. Furthermore, initiation of the treatment may have been influenced by clinical judgement and recent literature implying a possible link between GLP1 Ras use and pancreatitis or pancreatic cancer, introducing potential prescription bias. In addition, the relatively recent and rapidly increasing widespread use of GLP-1 receptor agonists may limit the ability to assess long-term effects on IPMN evolution. As a result, potential late modifications or clinically relevant changes may not yet be detectable within the available follow-up period.

Therefore, the results should be interpreted as hypothesis-generating. Despite these limitations, this study addresses an important gap in the current literature and may serve as a basis for future prospective, multicentre studies.

## Supporting information

S1 File List keywords. (DOCX)

S2 File REDCap database. (PDF)

S3 File Schedule for enrolment. (DOC)

S4 File General consent. (PDF)

## Abbreviations

IPMNs: Intraductal papillary mucinous neoplasms
GLP-1 RAs: Glucagon-like peptide-1 receptor agonists
AP: Acute pancreatitis
HFR: Fribourg Cantonal Hospital, Switzerland
HUG: Hopitaux universitaires Geneve, Switzerland
CER-VD: Commission cantonale d’éthique de la recherche sur l’être humain
