# Supplementary material for: Impact of GLP-1 receptor agonists on patients with intraductal papillary mucinous neoplasms: Study protocol for a multicentric cohort study
Source: PLoS One. 2026 Sep 9;21(9):e0357577. doi: 10.1371/journal.pone.0357577 (PMC13557380; doi:10.1371/journal.pone.0357577)
Supplement: Supplementary file 1 [file pone.0357577.s002.pdf]

Demographics

Record ID	__________________________________
Recruting Institution	◯ HFR Fribourg<br>◯ Inselspital Bern<br>◯ HUG Genève
Patient Identification Number (PID)	__________________________________
Patient Date Of Birth	__________________________________
Gender	◯ Male<br>◯ Female<br>◯ Other
Weight (kg)	__________________________________
Height (cm)	__________________________________
BMI	__________________________________
Smoking history	◯ Yes<br>◯ No
Alcohol history	◯ Yes<br>◯ No
Family history for pancreatic diseases	◯ Yes<br>◯ No<br>◯ Unknown
Positive family history	◯ Benign<br>◯ Malignant
IPMN ?	◯ Yes<br>◯ No
Date of IPMN diagnosis	__________________________________
Age at IPMN diagnosis	__________________________________
GLP1-RA ?	◯ Yes<br>◯ No
Indication for GLP1-RA	◯ Diabetes<br>◯ Obesity

GLP1-RA treatment (name)	◯ Semaglutide (Ozempic®, Rybelsus®, Wegovy®)<br>◯ Liraglutide (Victoza®, Saxenda®)<br>◯ Dulaglutide (Trulicity®)<br>◯ Exenatide (Byetta®, Bydureon®)<br>◯ Lixisenatide (Lyxumia®)<br>◯ Tirzepatide (Mounjaro®: Dual GLP-1/GIP Receptor Agonist)
Start of GLP1-RA treatment	______________________
GLP1-RA treatment dosis	______________________<br>((ex. OZEMPIC DualDose 2 mg/1.5ml, 0.5 mg/week))
Comorbidities	◯ Diabetes mellitus Type 2<br>◯ Pre-diabetes<br>◯ Dyslipidaemia<br>◯ Hypertension
Diabetes treatment	◯ Requiring insulin<br>◯ Non-requiring insulin
Other diabetes treatments (name of treatment and dosis)	______________________<br>((ex. METFORMIN 500 mg 1-0-0-0, JARDIANCE 10 mg 1-0-0-0))
Insulin treatment (name of treatment and dosis)	______________________<br>((ex. INSULIN NovoRapid according to scheme, Insuline Lantus 8-0-0-16 U))
Dyslipidemia treatment (name of treatment and dosis)	______________________<br>((ex. ATORVASTATIN 10 mg 0-0-1-0))
Hypertension treatment (name of treatment and dosis)	______________________<br>((ex. LISINOPRIL 5 mg 1-0-0-0))

IRM

Record ID ______________________

Date of aquisition ______________________

Age at IRM ______________________

Magnetic field ______________________

IPMN number
- ◯ 1
- ◯ 2
- ◯ 3
- ◯ 4
- ◯ 5
- ◯ >5

IPMN size Length (L) - craniocaudal (CC) ______________________ (unit of measure mm)

IPMN size Width (W) - transverse (TR) ______________________ (unit of measure mm)

IPMN size Depth (D) - anteroposterior (AP) ______________________ (unit of measure mm)

IPMN location
- ◯ Pancreatic head
- ◯ Pancreatic body
- ◯ Pancreatic queue
- ◯ other

Location other ______________________

Morphology ______________________

Principal conduit communication ______________________

Wall ______________________

Content ______________________

Nodule
- ◯ Yes
- ◯ No

Solid component	◯ Yes<br>◯ No
Calcification	◯ Yes<br>◯ No
Diffusion restriction	◯ Yes<br>◯ No
Enhancement	◯ Yes<br>◯ No
Growth	◯ Yes<br>◯ No
IPMN high risk stigmata as defined by international consensus guidelines	◯ Yes<br>◯ No
Presence of one or multiple of the following IPMN high risk stigmata	☐ Obstructive jaundice in a patient with cystic lesion of the head of the pancreas<br>☐ Enhancing mural nodule > 5 mm or solid component<br>☐ Main pancreatic duct > 10 mm<br>☐ Suspicious or positive results of cytology (if it was performed)
IPMN worrisome features as defined by international consensus guidelines	◯ Yes<br>◯ No
Presence of one or multiple of the following IPMN worrisome features	☐ Cyst > 30 mm<br>☐ Enhancing mural nodule < 5 mm<br>☐ Thick-ened/enhancing cyst walls<br>☐ Main pancreatic duct >5 mm and < 10 mm<br>☐ Abrupt change in caliber of pancreatic duct with distal pancreatic atrophy<br>☐ Lymphadenopathy<br>☐ Cystic growth rate > 2.5 mm/year
Pancreas trophicity	______________________
Ductal morphology	☐ Main duct (MD-IPMN)<br>☐ Branch duct (BD-IPMN)<br>☐ Mixed type (MT-IPMN)
Ductal caliber (mm)	______________________
Incidental additional pancreatic lesions	◯ Yes<br>◯ No
Description of incidental pancreatic lesions	______________________
Pancreatic parenchymal abnormalities	◯ Yes<br>◯ No
Description of pancreatic parenchymal abnormalities	______________________

Extrapancreatic findings ______________________________

eUS

Record ID	__________________________________
Date of eUS	__________________________________
Age at eUS	__________________________________
Cytopunction	◯ Yes<br>◯ No
Cytopunction histopatology reference	__________________________________<br>(example: Promed P2024-ABCD)
Malignancy in cytopunction	◯ Yes<br>◯ No
Confirmation of IPMN diagnosis in cytopunction	◯ Yes<br>◯ No

Lab values

Record ID

____

Date of lab

____

Age at lab

____

CRP (mg/l)

____

Leucocytes (G/l)

____

Lipase (U/l)

____

Amylase totale (U/l)

____

CEA (ng/ml)

____

CA 19-9 (U/ml)

____

Hémoglobine glyquée (%)

____

Fasting glucose (mmol/l)

____

Plasmatic insuline (µIU/mL)

____

HOMA index

____
(= (Fasting insulin, uIU/mL)*(Fasting glucose, mmol/L) / 22.5)

C peptide (pmol/l)

____

Follow-up, surgery and acute pancreatitis

Record ID

____

Change in follow-up protocol following introduction of GLP-1RA
- ◯ Yes
- ◯ No

Date of change in follow-up

____

Change in type of follow-up
- ◯ Increase in timing of follow-up (ex. from 1x/year to 1x/6months)
- ◯ Decrease in timing of follow-up (ex. from 1x/year to 1x/2 years)
- ◯ Surgical resection
- ◯ Other

Date of surgical resection

____

Age at op

____

Description of surgical resection and reconstruction

____
((ex. Pancreaticoduodenectomy (Whipple procedure) with Pancreaticojejunostomy, hepaticojejunostomy and gastrojejunostomy))

Histopathology

____

Description of other change in follow-up

____

Acute pancreatitis
- ◯ Yes
- ◯ No

Date of acute pancreatitis diagnosis

____

Age at diagnosis of AP

____

mCTSI Pancreatic inflammation
- ◯ 0 Normal pancreas
- ◯ 2 Intrinsic pancreatic abnormalities with or without inflammatory changes in peripancreatic fat
- ◯ 4 Pancreatic or peripancreatic fluid collection or fat necrosis

mCTSI Pancreatic Necrosis
- ◯ 0 None
- ◯ 2 ≤30% necrosis
- ◯ 4 >30% necrosis

mCTSI Extrapancreatic Complications	◯ 0 None<br>◯ 2 Presence of one or more (e.g., pleural effusion, ascites, vascular complications, GI tract involvement, or extrapancreatic parenchymal necrosis)
Discontinuation of GLP-1RA due to acute pancreatitis	◯ Yes<br>◯ No
