# Supplementary material for: Impact of GLP-1 receptor agonists on patients with intraductal papillary mucinous neoplasms: Study protocol for a multicentric cohort study
Source: PLoS One. 2026 Sep 9;21(9):e0357577. doi: 10.1371/journal.pone.0357577 (PMC13557380; doi:10.1371/journal.pone.0357577)
Supplement: Supplementary file 2 [file pone.0357577.s004.pdf]

hôpital fribourgeois
freiburger spital

Participer à la recherche médicale

Un consentement général et unique pour l'utilisation de vos données de santé et de vos échantillons à des fins de recherche.

Madame, Monsieur,

Vous pouvez jouer un rôle important pour l'avenir de la médecine. En effet, notre capacité à diagnostiquer et à traiter les maladies a considérablement progressé au cours des dernières décennies. Ces progrès ont été possibles grâce à l'effort soutenu de la recherche médicale à laquelle plusieurs générations de médecins, scientifiques et patients ont activement participé.

Une part importante de cette recherche repose sur l'utilisation des données cliniques des patients figurant dans les dossiers médicaux, tels que les résultats d'analyses de laboratoire, les traitements médicaux ou les prédispositions génétiques. Tout matériel biologique collecté durant le séjour hospitalier et qui n'est plus nécessaire pour les soins (sang, urine, échantillons de tissus, par exemple) peut être extrêmement précieux pour la recherche.

Ce document vous explique comment vous pouvez, vous aussi, contribuer aux progrès de la médecine, et vous fournit des explications sur la protection de vos données et sur vos droits.

Comment pouvez-vous contribuer à la recherche ?

Vous pouvez contribuer à la recherche en acceptant que vos données et échantillons biologiques résiduels soient conservés, transmis et réutilisés à des fins de recherche. Les données et échantillons incluent ceux qui ont été collectés par le passé. Ils comprennent aussi ceux qui seront collectés pour vos soins durant vos séjours et consultations actuels et futurs à l'hôpital fribourgeois (HFR).

Votre consentement est volontaire.

Il reste valable pour une durée indéfinie ou jusqu'à un éventuel retrait. Vous pouvez retirer votre consentement en tout temps sans avoir à justifier votre décision. Pour cela, il suffit que vous en informiez la direction médicale de l'HFR dont vous trouverez les coordonnées à la fin de ce document.

Si vous décidez de ne pas participer à la recherche en cochant « NON » au point A, vos données cliniques et échantillons biologiques ne pourront pas être utilisés pour la recherche. Si vous ne signez pas le formulaire de consentement, la loi prévoit que les échantillons et données peuvent être utilisés à titre exceptionnel pour la recherche après autorisation par la commission d'éthique compétente. Il est donc important pour vous d'exprimer votre choix.

Votre décision n'a aucun effet sur votre traitement médical.

Que se passe-t-il si vous retirez votre consentement ?

Dans ce cas, vos données et échantillons destinés à la recherche sont détruits, sous réserve des exigences légales. Ils ne sont dès lors plus disponibles pour de nouveaux projets de recherche.

Comment vos données de santé et vos échantillons biologiques sont-ils protégés ?

Les données sont enregistrées à l'hôpital et protégées dans le respect des exigences légales en vigueur[1]. Seuls les collaborateurs autorisés de l'hôpital, des médecins en charge de vos soins par exemple, ont accès à vos données et échantillons sous forme identifiée.

Vos échantillons biologiques sont stockés dans des biobanques. Celles-ci assurent la bonne gestion des échantillons et leur lien avec les données contenues dans votre dossier médical. Ces échantillons et ces données peuvent être utilisés pour votre prise en charge médicale, ainsi qu'à des fins de recherche. Elles sont soumises à des normes de sécurité et de qualité (www.h-fr.ch/nos-recherches/consentement-general).

Si vos données et échantillons sont utilisés pour un projet de recherche, ils seront codés ou anonymisés.

- Le terme « codé » signifie que toutes les informations personnelles (par exemple votre nom ou votre date de naissance) sont remplacées par un code. La clé qui permet de savoir quel code correspond à quel individu est conservée en toute sécurité par une personne qui n'est pas impliquée dans le projet de recherche. Les personnes qui ne possèdent pas la clé de codage ne sont pas en mesure de vous identifier.
- Le terme « anonymisé » signifie que le lien entre le matériel biologique ou les données associées et l'individu est définitivement rompu. Selon la loi, des données sont dites anonymisées lorsqu'elles ne peuvent pas être mises en relation avec une personne déterminée sans engager des efforts démesurés. En principe il n'est plus possible d'identifier la personne concernée, même si une anonymisation absolue ne peut pas être garantie. Une fois les données et les échantillons anonymisés, la personne concernée ne peut plus retirer son consentement pour l'utilisation de ces données et échantillons. Elle ne peut pas non plus être informée des éventuels résultats de recherche pertinents pour sa santé. De même, les échantillons ou données anonymisés ne seront pas détruits en cas de retrait du consentement.

La majorité des projets de recherche utilisent des données codées, en particulier lorsqu'ils peuvent générer des résultats pertinents pour la santé des personnes concernées.

Les droits relatifs à la protection de vos données dans le cadre de la recherche sont les mêmes que dans le cadre des soins, notamment le droit d'accéder à vos données personnelles.

Qui peut utiliser vos données de santé et échantillons ?

Les données et échantillons peuvent être utilisés par des chercheurs ayant reçu une autorisation de la Commission d'éthique de la recherche compétente. Les projets de recherche sont menés à l'HFR ou en collaboration avec d'autres institutions publiques (d'autres hôpitaux ou universités, par exemple) et des entités privées (des compagnies pharmaceutiques, par exemple) en Suisse ou à l'étranger.

La transmission de données ou d'échantillons à l'étranger à des fins de recherche n'est possible que si les conditions de protection des données dans le pays de destination sont au moins équivalentes à celles appliquées en Suisse.

Les projets peuvent inclure des analyses génétiques à des fins de recherche. Tout projet de recherche utilisant vos données ou échantillons doit avoir obtenu l'autorisation de la Commission d'éthique de la recherche compétente.

[1] En particulier, la loi sur la recherche sur l'être humain et la législation sur la protection des données.

Serez-vous informé-e des résultats de recherche ?

La recherche menée avec vos échantillons et données ne révélera en principe aucune information individuelle pour votre santé. Dans de rares cas, il pourrait toutefois arriver que des résultats pertinents, pour lesquels des traitements ou des actions de prévention sont disponibles, soient découverts. Dans ce cas, vous en seriez informé-e.

Si vous ne souhaitez pas recevoir une telle information, veuillez-vous adresser à la direction médicale de l'hôpital fribourgeois dont vous trouverez les coordonnées à la fin de ce document.

Votre participation engendre-t-elle des frais ou des bénéfices financiers ?

Votre participation n'engendre aucun frais supplémentaire pour vous ou votre assurance. La loi exclut la commercialisation des données et des échantillons. Ainsi, aucun avantage financier ne sera généré pour vous ou pour l'hôpital.

Vous pouvez nous communiquer votre décision en remplissant et en signant la déclaration de consentement.

La déclaration de consentement comprend trois étapes :

A. après avoir noté votre nom, prénom et date de naissance, indiquez si vous acceptez ou refusez l'utilisation de vos données de santé et échantillons à des fins de recherche.

B. signez et datez la déclaration, afin de confirmer votre décision.

C. Lorsque vous avez complété la déclaration de consentement, vous pouvez soit la remettre à un des secrétariats de l'hôpital soit la renvoyer avec une copie de pièce d'identité (carte d'identité ou passeport) à l'adresse indiquée au dos de ce document.

Si vous avez des questions ou si vous souhaitez retirer votre consentement, n'hésitez pas à nous contacter.

Par courrier :
HFR - hôpital fribourgeois
Direction médicale
Case postale
1708 Fribourg

Par e-mail :
direction.medicale@h-fr.ch

Par téléphone :
Direction médicale
T +41 26 306 01 60
Lu - Ve : 08h30 - 11h30 / 13h30 - 16h30

Informations complémentaires
www.h-fr.ch/nos-recherches/consentement-general

hôpital fribourgeois
freiburger spital

Étiquette patient

Déclaration de consentement pour l'utilisation des données de santé et des échantillons à des fins de recherche

....

Nom et prénom | Date de naissance

A. J'accepte que mes données de santé et mes échantillons biologiques résiduels collectés durant les soins (consultations ambulatoires et hospitalisations) soient conservés, transmis et utilisés à des fins de recherche.

☐ oui ☐ non

Quelle que soit votre réponse, veuillez passer au point B.

B. Confirmation de ma décision

Je confirme m'être vu offrir ou avoir eu un contact direct avec un professionnel de la santé afin de répondre à toutes mes questions relatives au présent document et j'ai compris:

- les explications sur la réutilisation de mes données de santé et échantillons biologiques à des fins de recherche, détaillées dans la brochure d'information;
- que mes données et échantillons sont protégés et qu'ils ne seront utilisés pour la recherche que de manière codée ou anonymisée;
- que mes données et échantillons peuvent être utilisés dans des projets de recherche nationaux et internationaux, dans les secteurs public et privé ;
- que les projets peuvent inclure des analyses génétiques sur mes échantillons ;
- que je pourrais être recontacté-e dans le cas où des résultats pertinents pour ma santé seraient mis en évidence ;
- que ma décision est volontaire et n'a pas d'effet sur mon traitement médical ;
- que ma décision est valable pour une durée illimitée à moins que je retire mon consentement ;
- que je peux retirer mon consentement à n'importe quel moment sans avoir à justifier ma décision ;
- que si je coche « NON » au point A en signant cette déclaration, mes données cliniques et échantillons biologiques ne pourront pas être utilisés pour la recherche ;
- que si je ne signe pas la déclaration de consentement la loi prévoit que mes données et échantillons pourront exceptionnellement être utilisés si la commission d'éthique compétente donne son autorisation spéciale.

....

Lieu et date | Signature du/de la patient-e

En cas de question ou de remarque, n'hésitez pas à nous contacter.

Par courrier :
HFR - hôpital fribourgeois
Direction médicale
Case postale
1708 Fribourg

Par téléphone :
Direction médicale
T +41 26 306 01 60
Lu - Ve : 08h30 - 11h30 / 13h30 - 16h30

Par e-mail :
direction.medicale@h-fr.ch

Informations complémentaires
www.h-fr.ch/nos-recherches/consentement-general

hôpital fribourgeois
freiburger spital

Teilnahme an medizinischer Forschung

Einmalige allgemeine Einverständniserklärung (Generalkonsent) zur Verwendung Ihrer Gesundheitsdaten und Proben für Forschungszwecke

Sehr geehrte Damen und Herren

Sie können für die Zukunft der Medizin eine wichtige Rolle spielen. In der Tat hat sich unsere Fähigkeit, Krankheiten zu diagnostizieren und zu behandeln, in den letzten Jahrzehnten erheblich verbessert. Dieser Fortschritt war möglich dank der anhaltenden Anstrengungen der medizinischen Forschung, an der mehrere Generationen von Ärzten, Wissenschaftlern und Patienten aktiv mitgewirkt haben.

Ein wichtiger Teil dieser Forschung stützt sich auf die Verwendung von klinischen Daten aus Patientendossiers, wie z. B. Laborergebnisse, medizinische Behandlungen oder genetische Veranlagungen. Jegliches während des Spitalaufenthalts gesammelte biologische Material, das nicht mehr für die Pflege benötigt wird (z. B. Blut, Urin, Gewebeproben), kann für die Forschung äusserst wertvoll sein.

Dieses Dokument erklärt, wie auch Sie zum medizinischen Fortschritt beitragen können, und liefert Informationen zum Datenschutz und zu Ihren Rechten.

Wie können Sie zur Forschung beitragen?

Sie können zur Forschung beitragen, indem Sie der Speicherung, Übertragung und Verwendung Ihrer Daten und biologischen Proben zu Forschungszwecken zustimmen. Zu den Daten und Proben gehören auch solche, die in der Vergangenheit gesammelt wurden. Dazu gehören auch diejenigen, die während Ihrer derzeitigen und zukünftigen Aufenthalte und Konsultationen im freiburger spital (HFR) im Rahmen Ihrer Betreuung gesammelt werden.

Ihre Zustimmung ist freiwillig.

Sie bleibt auf unbestimmte Zeit oder bis zu einem möglichen Rückzug gültig. Sie können Ihre Zustimmung jederzeit zurückziehen, ohne dass Sie Ihre Entscheidung begründen müssen. Dazu genügt es, die Medizinische Direktion des HFR zu informieren, deren Kontaktdaten am Ende dieses Dokuments aufgeführt sind.

Wenn Sie nicht zur Forschung beitragen möchten und unter Punkt A „Nein" ankreuzen, können Ihre klinischen Daten und biologischen Proben nicht für Forschungszwecke verwendet werden. Wenn Sie die Einverständniserklärung nicht unterzeichnen, sieht das Gesetz vor, dass die Proben und Daten ausnahmsweise für die Forschung verwendet werden dürfen, sofern die zuständige Ethikkommission dies genehmigt. Es ist daher wichtig, dass Sie Ihre Entscheidung klar festhalten.

Ihr Entscheid hat keinerlei Auswirkungen auf Ihre medizinische Betreuung.

Was passiert, wenn Sie Ihre Zustimmung zurückziehen?

In diesem Fall werden Ihre für die Forschung bestimmten Daten und Proben, vorbehaltlich der gesetzlichen Bestimmungen, vernichtet. Sie stehen daher nicht mehr für neue Forschungsprojekte zur Verfügung.

Wie werden Ihre Gesundheitsdaten und biologischen Proben geschützt?

Die Daten werden im Spital gespeichert und gemäss den geltenden gesetzlichen Bestimmungen geschützt[1]. Nur autorisiertes Spitalpersonal, z. B. die für Ihre Behandlung zuständigen Ärzte, haben in identifizierter Form Zugang zu Ihren Daten und Proben.

Ihre biologischen Proben werden in Biobanken gelagert. Diese gewährleisten die ordnungsgemässe Verwaltung der Proben und ihre Verknüpfung mit den Daten in Ihrem Patientendossier. Diese Proben und Daten können sowohl für Ihre medizinische Versorgung als auch für Forschungszwecke verwendet werden. Sie unterliegen Sicherheits- und Qualitätsstandards (www.h-fr.ch/de/forschung/einverstaendniserklaerung).

Wenn Ihre Daten und Proben für ein Forschungsprojekt verwendet werden, werden sie verschlüsselt oder anonymisiert.

- Der Begriff „verschlüsselt" bedeutet, dass alle persönlichen Informationen (z. B. Ihr Name oder Ihr Geburtsdatum) durch einen Code ersetzt werden. Der Codierungsschlüssel, mit dem ermittelt werden kann, welcher Code welcher Person entspricht, wird von jemandem, der nicht am Forschungsprojekt beteiligt ist, sicher aufbewahrt. Personen, die nicht im Besitz des Codierungsschlüssels sind, können Sie nicht identifizieren.
- „Anonymisiert" bedeutet, dass die Verbindung zwischen dem biologischen Material oder den zugehörigen Daten und dem Individuum dauerhaft getrennt wird. Nach dem Gesetz gelten Daten als anonym, wenn sie nicht ohne unverhältnismässigen Aufwand mit einer bestimmten Person in Verbindung gebracht werden können. Grundsätzlich ist es nicht mehr möglich, die betroffene Person zu identifizieren, auch wenn eine absolute Anonymität nicht garantiert werden kann. Sobald die Daten und Proben anonymisiert wurden, kann die betroffene Person ihre Zustimmung zur Verwendung dieser Daten und Proben nicht mehr zurücknehmen. Sie kann auch nicht über Forschungsergebnisse informiert werden, die für ihre Gesundheit relevant sind. Ebenso werden Proben oder anonymisierte Daten im Falle eines Widerrufs der Zustimmung nicht vernichtet.

Die Mehrzahl der Forschungsprojekte verwendet codierte Daten, insbesondere wenn sie gesundheitsrelevante Ergebnisse für die beteiligten Personen liefern können.

In Bezug auf den Schutz Ihrer Daten im Rahmen der Forschung gelten die gleichen Rechte wie im Rahmen Ihrer Betreuung, insbesondere das Recht auf Zugang zu Ihren persönlichen Daten.

Wer kann Ihre Gesundheitsdaten und Proben verwenden?

Die Daten und Proben können von Forschern verwendet werden, welche die Genehmigung der zuständigen Forschungsethikkommission erhalten haben. Forschungsprojekte werden am HFR oder in Zusammenarbeit mit anderen öffentlichen Institutionen (z. B. anderen Spitälern oder Universitäten) und privaten Einrichtungen (z. B. Pharmaunternehmen) in der Schweiz oder im Ausland durchgeführt.

Die Übermittlung von Daten oder Proben ins Ausland zu Forschungszwecken ist nur möglich, wenn die Datenschutzbedingungen im Zielland mindestens gleichwertig zu denen in der Schweiz sind.

Die Projekte können genetische Analysen für Forschungszwecke umfassen. Jedes Forschungsprojekt, bei dem Ihre Daten oder Proben verwendet werden, muss die Genehmigung der zuständigen Forschungsethikkommission haben.

[1] Insbesondere das Humanforschungsgesetz und die Datenschutzgesetzgebung.

Werden Sie über die Forschungsergebnisse informiert?

Die mit Ihren Proben und Daten durchgeführte Forschung wird in der Regel keine individuellen Informationen zu Ihrer Gesundheit ergeben. In seltenen Fällen können jedoch in Bezug auf Behandlungs- oder Präventionsmassnahmen relevante Ergebnisse ermittelt werden. In diesem Fall werden Sie entsprechend informiert.

Wenn Sie keine solchen Informationen erhalten möchten, wenden Sie sich bitte an die Medizinische Direktion des freiburger spitals, deren Kontaktdaten am Ende dieses Dokuments aufgeführt sind.

Bringt Ihre Teilnahme finanzielle Kosten oder Vorteile mit sich?

Durch Ihre Teilnahme entstehen Ihnen oder Ihrer Versicherung keine zusätzlichen Kosten. Das Gesetz schliesst die Kommerzialisierung von Daten und Proben aus. Es entsteht also kein finanzieller Nutzen für Sie oder das Spital.

Sie können uns Ihre Entscheidung mitteilen, indem Sie die Einverständniserklärung ausfüllen und unterschreiben.

Die Einverständniserklärung umfasst drei Schritte:

A. Nachdem Sie Ihren vollständigen Namen und Ihr Geburtsdatum notiert haben, geben Sie an, ob Sie mit der Verwendung Ihrer Gesundheitsdaten und Proben für Forschungszwecke einverstanden sind oder nicht.

B. Unterschreiben und datieren Sie die Erklärung, um Ihre Entscheidung zu bestätigen.

C. Wenn Sie die Einverständniserklärung ausgefüllt haben, können Sie sie entweder bei einem der Sekretariate des HFR abgeben oder zusammen mit einer Kopie eines Ausweisdokuments (Personalausweis oder Reisepass) an die auf der Rückseite dieses Dokuments angegebene Adresse zurücksenden.

Wenn Sie Fragen haben oder Ihre Zustimmung zurückziehen möchten, kontaktieren Sie uns bitte.

Per Post:
freiburger spital (HFR)
Medizinische Direktion
Postfach
1708 Freiburg

Telefonisch:
Medizinische Direktion
T +41 26 306 01 60
Mo–Fr: 8.00 - 11.30 / 13.30 - 16.30 Uhr

Per E-Mail:
direction.medicale@h-fr.ch

Zusätzliche Informationen
www.h-fr.ch/de/forschung/einverstaendniserklaerung

hôpital fribourgeois
freiburger spital

Patientenetikett

Einverständniserklärung zur Verwendung von Gesundheitsdaten und Proben für Forschungszwecke

....

Name und Vorname — Geburtsdatum

A. Ich bin damit einverstanden, dass meine Gesundheitsdaten und die während der Behandlung (ambulante Konsultationen und Spitalaufenthalte) gesammelten biologischen Proben gespeichert, übermittelt und zu Forschungszwecken verwendet werden dürfen.

☐ Ja ☐ Nein

Unabhängig von Ihrer Antwort gehen Sie zu Punkt B.

B. Bestätigung meiner Entscheidung

Ich bestätige, dass mir angeboten wurde oder ich direkten Kontakt mit einer Gesundheitsfachperson hatte, um Fragen zu diesem Dokument zu beantworten, und ich verstehe:

- die Erläuterungen zur Wiederverwendung meiner Gesundheitsdaten und biologischen Proben für Forschungszwecke, die in der Informationsbroschüre beschrieben sind;
- dass meine Daten und Proben geschützt sind und nur zu Forschungszwecken in verschlüsselter oder anonymisierter Form verwendet werden;
- dass meine Daten und Proben in nationalen und internationalen Forschungsprojekten im öffentlichen und privaten Sektor verwendet werden können;
- dass die Projekte eine genetische Analyse meiner Proben beinhalten können;
- dass ich wieder kontaktiert werden könnte, falls sich relevante Gesundheitsergebnisse ergeben;
- dass meine Entscheidung freiwillig ist und keine Auswirkungen auf meine medizinische Behandlung hat;
- dass meine Entscheidung für einen unbegrenzten Zeitraum gültig ist, es sei denn, ich ziehe meine Zustimmung zurück;
- dass ich meine Zustimmung jederzeit zurückziehen kann, ohne meine Entscheidung begründen zu müssen;
- dass, wenn ich bei Punkt A „Nein" ankreuze, meine Gesundheitsdaten und biologischen Proben nicht für die Forschung verwendet werden;
- dass, wenn ich die Einwilligungserklärung nicht unterschreibe, das Gesetz vorsieht, dass meine Daten und Proben ausnahmsweise verwendet werden dürfen, wenn die zuständige Ethikkommission eine Sondergenehmigung erteilt.

....

Ort und Datum — Unterschrift der Patientin/des Patienten

Bei Fragen oder Anmerkungen zögern Sie nicht, uns zu kontaktieren.

Per Post:
freiburger spital (HFR)
Medizinische Direktion
Postfach
1708 Freiburg

Telefonisch:
Medizinische Direktion
T +41 26 306 01 60
Mo–Fr: 8.00 - 11.30 / 13.30 - 16.30 Uhr

Per E-Mail:
direction.medicale@h-fr.ch

Zusätzliche Informationen
www.h-fr.ch/de/forschung/einverstaendniserklaerung

Déclaration de consentement pour l’utilisation des données de santé et des échantillons à des fins de recherche

J’accepte que mes données de santé et mes échantillons biologiques collectés durant les soins (consultations ambulatoires et hospitalisations) soient utilisés à des fins de recherche

Oui ☐ Non ☐

J’ai compris :

- les explications sur la réutilisation de mes données de santé et échantillons biologiques à des fins de recherche, détaillées dans l’information ci-dessus.
- que mes données personnelles sont protégées.
- que mes données et échantillons biologiques peuvent être utilisés dans des projets de recherche nationaux et internationaux, dans les secteurs public et privé.
- que les projets peuvent inclure des analyses génétiques sur mes échantillons, à des fins de recherche.
- que je peux être recontacté-e si des résultats pertinents me concernant sont mis en évidence.
- que ma décision est libre et n’a pas d’effet sur mon traitement médical.
- que ma décision est valable pour une durée illimitée.
- que je peux retirer mon consentement à tout moment sans avoir à justifier ma décision.

Nom, prénom, date de naissance
ou étiquette patient

Lieu, date — Signature du patient, si capable de discernement

---

Lieu, date — Signature du représentant légal, si nécessaire (nom et relation avec le/la patient-e)

Si vous avez des questions, ou si vous souhaitez recevoir une copie de ce formulaire avec signature, prenez contact avec le **Centre de Recherche Clinique**
rue Gabrielle-Perret-Gentil 4, 1211 Genève 14
022 372 91 57, aider-la-recherche@hcuge.ch

Einwilligungserklärung zur Weiterverwendung von gesundheitsbezogenen Daten und Proben und zur Blutentnahme

1. Ich stimme hiermit zu,

dass meine **gesundheitsbezogenen Daten und Proben**, die während meines ambulanten oder stationären Spitalaufenthaltes erhoben bzw. entnommen werden, der Forschung zur Verfügung stehen.

☐ Ja ☐ Nein

2. Ich stimme hiermit zu,

- dass mir zu Forschungszwecken Blut (maximal 20 Milliliter, entspricht zwei Entnahmeröhrchen) entnommen werden darf
- dass bei Bedarf zusammen mit der Blutentnahme folgende zusätzlichen Angaben erhoben werden dürfen: Grösse / Gewicht / Ethnie (Abstammung, z.B. Kaukasisch)
- dass ich mit der Nutzung von biologischem Material und gesundheitsbezogenen Daten gemäss Teil 1) einverstanden bin

☐ Ja ☐ Nein

Ich weiss, dass

- auf dem Informationsblatt (V3.0 August 2020) die Bestimmungen zur Weiterverwendung meiner Daten und Proben beschrieben sind. Ich habe diese Informationen gelesen und verstanden.
- meine persönlichen Daten geschützt sind.
- meine Daten und Proben in nationalen und internationalen Projekten, innerhalb öffentlicher und privater Institutionen, verwendet werden können.
- meine Proben zu Forschungszwecken für genetische Analysen verwendet werden können.
- ich kontaktiert werden kann, falls für mich relevante Informationen gefunden werden.
- meine Entscheidung freiwillig ist und keinen Einfluss auf meine Behandlung hat.
- meine Entscheidung zeitlich unbegrenzt gilt.
- ich meine Zustimmung jederzeit ohne Begründung zurückziehen kann.
- das Reglement der Liquid Biobank Bern jederzeit unter www.biobankbern.ch einsehbar ist.

Ort, Datum	Unterschrift Patient/in, sofern urteilsfähig
Ort, Datum	Unterschrift gesetzliche/r Vertreter/in, falls zutreffend (Name und Bezug zu Patient/in)

Sie erhalten eine Kopie dieser Seite mit Unterschrift, wenn Sie dies wünschen. Bei Fragen wenden Sie sich bitte an Ihre behandelnden Ärzte oder an folgenden Kontakt:

Insel Gruppe AG, Direktion Lehre und Forschung CH-3010 Bern
einwilligungserklaerung-forschung@insel.ch, www.inselgruppe.ch/generalconsent
Telefon: 031 632 58 45

Information zur Weiterverwendung gesundheitsbezogener Daten und Proben zu Forschungszwecken

Sehr geehrte Patientin, sehr geehrter Patient

Die Erkennung und Behandlung von Krankheiten hat in den letzten Jahrzehnten enorme Fortschritte gemacht. Diese Entwicklung ist das Ergebnis langjähriger Forschung, an der Ärzte, Wissenschaftler und Patienten verschiedener Generationen aktiv beteiligt sind.

Die Forschung ist dabei wesentlich auf Daten aus Krankengeschichten von Patientinnen und Patienten angewiesen. Diese können zum Beispiel Ergebnisse aus Laboranalysen, Informationen zu einer Therapie oder über erbliche Veranlagungen zu bestimmten Krankheiten umfassen. Auch biologisches Material, das als Probe während eines Spitalaufenthaltes entnommen wurde und für Diagnosezwecke nicht mehr benötigt wird, ist sehr wertvoll für die Forschung. Bei diesen übriggebliebenen Proben kann es sich beispielsweise um Blut-, Urin- oder Gewebeproben handeln.

Diese Information erklärt, wie Sie als Patientin oder Patient zum medizinischen Fortschritt beitragen können und zeigt auf, wie Ihre Patientendaten und -rechte geschützt werden. Wir danken Ihnen herzlich für Ihr Interesse und Ihre Unterstützung.

Wie können Sie Ihren Beitrag zur Forschung leisten?

Wenn Sie das Einwilligungsformular mit «Ja» unterschreiben, stellen Sie Ihre gesundheitsbezogenen Daten und übriggebliebenen Proben der Forschung zur Verfügung. Die Einwilligung gilt für alle Daten, die bereits im Spital erhoben wurden oder zukünftig erhoben werden. Dies gilt im gleichen Sinne für die Proben.

Ihre Einwilligung ist freiwillig. Sie gilt zeitlich unbegrenzt, falls sie nicht widerrufen (zurückgezogen) wird. Sie können Ihre Einwilligung jederzeit über die untenstehende Kontaktadresse widerrufen, ohne eine Begründung dafür abzugeben. Nach dem Widerruf werden Ihre Daten und Proben für neue Forschungsprojekte nicht mehr zur Verfügung gestellt. Ihre Entscheidung hat keinen Einfluss auf Ihre medizinische Behandlung.

Wie werden Ihre gesundheitsbezogenen Daten und Proben geschützt?

Ihre Daten werden im Spital gemäss den gesetzlichen Vorschriften bearbeitet und geschützt. Nur befugte Mitarbeiter des Spitals, wie beispielsweise Ärzte, haben Einsicht in die unverschlüsselten Daten aus Ihrer Krankengeschichte und Zugang zu Ihren unverschlüsselten Proben. Ihre Proben sind in Biobanken verwahrt. Eine Biobank stellt eine strukturierte Sammlung verschiedener Proben unter bestimmten Sicherheitsbestimmungen (Biobankreglement) dar.

Wenn Ihre Daten und Proben für Forschungsprojekte verwendet werden, so werden diese verschlüsselt oder anonymisiert. Verschlüsselt bedeutet, dass alle personenbezogenen Daten wie Ihr Name oder Geburtsdatum durch einen Code ersetzt werden. Der Schlüssel, der zeigt, welcher Code zu welcher Person gehört, wird durch eine nicht am Forschungsprojekt beteiligte Person sicher verwahrt. Personen, die keinen Zugang zum Schlüssel haben, können Sie nicht identifizieren. Werden Daten und Proben anonymisiert, gibt es keinen Schlüssel, der auf Ihre Person zurückführt.

Wer darf Ihre gesundheitsbezogenen Daten und Proben verwenden?

Ihre Daten und Proben werden berechtigten Forschenden an unserem Spital für Forschungsprojekte zur Verfügung gestellt oder können in Forschungsprojekten in Zusammenarbeit mit anderen öffentlichen oder privaten Institutionen verwendet werden (andere Spitäler, Universitäten oder pharmazeutische Unternehmen). Die Projekte können in der Schweiz oder im Ausland durchgeführt werden und gegebenenfalls genetische Analysen beinhalten. Bei Forschungsprojekten im Ausland gelten mindestens die gleichen Anforderungen an den Datenschutz wie in der Schweiz. Forschungsprojekte unterliegen generell einer Überprüfung der zuständigen Ethikkommission.

Werden Sie über Forschungsresultate informiert?

Forschungsprojekte mit Daten und Proben führen in der Regel nicht zu Informationen, die unmittelbar für die Gesundheit einer einzelnen Person von Belang sind. Sollte dennoch ein für Sie bedeutendes Ergebnis gefunden werden und eine medizinische Massnahme verfügbar sein, ist eine Kontaktaufnahme durch das Spital möglich.

Werden Sie finanzielle Vor- oder Nachteile haben?

Es entstehen für Sie keine zusätzlichen Kosten. Es ist von Gesetzes wegen ausgeschlossen, mit Ihren Daten und Proben Geld zu erwirtschaften. Es entsteht daher weder für Sie noch für das Spital ein finanzieller Vorteil.

Falls Sie noch Fragen haben oder zusätzliche Informationen wünschen, wenden Sie sich bitte an die untenstehende Kontaktadresse oder besuchen Sie unsere Website.

Information zu einer Blutentnahme für die Liquid BioBank Bern

Für aussagekräftige Forschungsergebnisse sind Forschende darauf angewiesen, auf zahlreiche qualitativ hochwertige Proben zurückgreifen zu können. Aus diesem Grund ist es für die Insel Gruppe ausgesprochen wichtig von den Patienten eine zusätzliche Blutprobe zu entnehmen. Ihre Einwilligung zu dieser Blutentnahme geben Sie gesondert.

Wie wird Ihre Probe genutzt?

Für die Aufbewahrung und Weitergabe Ihrer Probe gelten dieselben Vorschriften wie für die gesundheitsbezogenen Informationen und medizinischen Proben. Insbesondere können Sie die Einwilligung jederzeit ohne Begründung verweigern oder widerrufen. Zusätzlich ist die Verwaltung und Weitergabe der Proben in einem von der kantonalen Ethikkommission bewilligten Reglement definiert.

Warum wird eine zusätzliche Blutentnahme benötigt?

Im Vergleich zu Untersuchungen, die für Ihre Behandlung durchgeführt werden, hat die Forschung andere Anforderungen an die Probenqualität. Insbesondere ist wichtig, dass alle in einer Studie verwendeten Proben auf die gleiche Art verarbeitet wurden.
Entnahme, Transport und Lagerung beeinflussen die Qualität einer Blutprobe. Dank der zusätzlichen Blutentnahme können diese Prozesse standardisiert und dokumentiert werden. Diese Qualitätssicherung und Lagerung werden in der Liquid BioBank Bern sichergestellt. Die Liquid BioBank Bern kann dadurch den verschiedenen Forschungsvorhaben Proben mit einheitlicher und gleichbleibender Qualität zur Verfügung stellen.

Warum sind zusätzliche Angaben (Grösse/Gewicht/Ethnie) erforderlich?

Ihre Blutprobe ist für die Forschung äusserst wertvoll. Neben den bereits vorhandenen gesundheitlichen Daten dienen die zusätzlichen Angaben dazu, Proben zu kategorisieren. So kann Ihre Probe denjenigen Forschungsvorhaben zur Verfügung gestellt werden, welche den grössten Nutzen daraus ziehen können.

Wann erfolgt die Blutentnahme?

Die Blutentnahme erfolgt im Rahmen Ihres Spitalaufenthaltes. Sie werden nicht speziell für eine BioBank-Blutentnahme eingeladen.

Was ist eine Biobank?

In einer sogenannten Biobank werden Proben gesammelt, die im Rahmen von Studien oder für die Forschung allgemein, angelegt werden. Zu jeder eingelagerten Probe werden Daten zur Probe und zum Spender selbst gespeichert.

Welchen Zweck verfolgt die Liquid BioBank Bern?

In den letzten Jahren wurden Proben meist gezielt für einzelne Studien gesammelt. Wertvolles Material von Probanden stand somit nur einem beschränkten Kreis von Forschenden zur Verfügung. Mit dem Aufbau der Liquid BioBank Bern sollen Proben gesammelt und verschiedenen Forschungsprojekten zur Verfügung gestellt werden. Dadurch wird der Nutzen von einzelnen Proben für die Forschung erhöht und der Aufwand für die medizinische Forschung gesenkt.

Wo finden Sie weitere Informationen zur Liquid BioBank Bern?

Die Liquid BioBank Bern (LBB) gehört der Insel Gruppe. Die LBB wurde eingerichtet, um insbesondere biomedizinische Forschung nachhaltig zu fördern und durch Zurverfügungstellung von Patientenproben mit im internationalen Vergleich herausragender Qualität zu unterstützen. Das Ziel ist es Prädiktion, Prävention, Diagnose und Therapie in der Humanmedizin zu verbessern. Ausserdem können mit den gesammelten Proben und Daten auch grundlegende Mechanismen zum Verständnis von Krankheiten erforscht werden.

Wenn Sie mehr über die Liquid BioBank Bern erfahren möchten, besuchen Sie unsere Website
www.biobankbern.ch
