# Supplementary material for: Impact of GLP-1 receptor agonists on patients with intraductal papillary mucinous neoplasms: Study protocol for a multicentric cohort study
Source: PLoS One. 2026 Sep 9;21(9):e0357577. doi: 10.1371/journal.pone.0357577 (PMC13557380; doi:10.1371/journal.pone.0357577)
Supplement: Supplementary file 3 [file pone.0357577.s001.docx]

**List A: Patients with IPMN**

1. ICD-10 Codes: The following ICD-10 diagnostic codes will be used to identify hospitalized patients with pancreatic cystic lesions compatible with IPMN:

- K86.2 — Cyst of pancreas
- D13.7 — Benign neoplasm of pancreas

2. Keywords: The following keywords will be used in medical reports, radiology reports, consultation notes, and pathology reports: IPMN, Intraductal Papillary Mucinous Neoplasm, Intraductal Papillary Mucinous Tumor, Intraductal mucinous neoplasm, Pancreatic cystic lesion, Pancreatic cystic neoplasm, Mucinous pancreatic cyst, Pancreatic cyst, Pancreatic mucinous lesion.

French version:

1. CIM-10

- K86.2 — Kyste du pancréas
- D13.7 — Tumeur bénigne du pancréas

2. Mots-clés: TIPMP, IPMN, Tumeur Intracanalaire Papillaire Mucineuse du Pancréas, Néoplasie papillaire mucineuse intracanalaire, Adénome mucineux intracanalaire, Tumeur mucineuse intracanalaire, Lésion kystique pancréatique, Lésion kystique du pancréas, Kyste mucineux pancréatique, Lésion pancréatique mucineuse.

German version:

1. ICD-10-Codes

- K86.2 — Pankreaszyste
- D13.7 — Gutartige Neubildung des Pankreas

2. Schlüsselwörter: IPMN, Intraduktale papillär-muzinöse Neoplasie, Intraductal Papillary Mucinous Neoplasm, Muzinöse Pankreaszyste, Zystische Pankreasläsion, Pankreaszyste, Muzinöse intraduktale Neoplasie.

**List B: Patients treated with GLP-1 Receptor Agonists**

Patients exposed to GLP-1 receptor agonists will be identified using following medication names, commercial names, and ATC medication codes:

- Semaglutide: Ozempic®, Wegovy®, Rybelsus® — A10BJ06, A08BX12, A10BJ06
- Dulaglutide: Trulicity® — A10BJ05
- Exenatide: Byetta®, Bydureon® — A10BJ01
- Liraglutide: Victoza®, Saxenda® — A10BJ02, A08AX07
- Lixisenatide: Lyxumia® — A10BJ03
- Tirzepatide: Mounjaro® — A10BK01
