# Supplementary material for: Impact of GLP-1 receptor agonists on patients with intraductal papillary mucinous neoplasms: Study protocol for a multicentric cohort study
Source: PLoS One. 2026 Sep 9;21(9):e0357577. doi: 10.1371/journal.pone.0357577 (PMC13557380; doi:10.1371/journal.pone.0357577)
Supplement: Supplementary file 4 [file pone.0357577.s006.docx]

**Response to Reviewers**

**Manuscript ID [PONE-D-25-43564]**

**Title:** *Impact of GLP-1 Receptor Agonists on Patients with Intraductal Papillary Mucinous Neoplasms: Study Protocol for a Multicentric Cohort Study*

**Reviewer #1**:

In the paper, Melissa et al presents a multicentric retrospective cohort study protocol. They aim to study the association between the usage of GLP-1 receptor agonist (GLP-1 RA) and the progression of intraductal papillary mucinous neoplasms (IPMNs). The topic is clinically relevant given the increasing prevalence of IPMNs and the widespread use of GLP-1 RAs for diabetes and obesity. The protocol is generally well written, follows SPIRIT 2013 guidelines, and clearly describes objectives, inclusion criteria, outcomes, and planned analyses. However, there are important methodological and interpretative limitations, particularly related to sample size, comparator definition, confounding control, and feasibility of the planned statistical analyses, that should be addressed or clarified to strengthen the protocol and avoid overinterpretation of results.

1. Expected sample size is too limited.

- Following the introduction of control groups, the expected total sample size has been increased ranging between approximately 30 and 60 patients. We acknowledge that this sample size is constrained by the rarity of concomitant GLP-1 receptor agonist exposure and IPMN diagnosis. However, the multicentric design of this retrospective study, involving three tertiary care centres, was specifically implemented to maximise patient identification and recruitment. Despite these efforts, we recognise that the study remains exploratory in nature and that the limited sample size represents an inherent limitation.
- Page 6-7, lines 243-281: *The sample size is expected to be between 10 and 20 patients for the condition with GLP-1 RAs and IPMN, due to the rare nature of the patient population with both IPMN and GLP-1 RA use. The other conditions (GLP-1 RA & no-IPMN, no GLP-1 RA & IPMN) will match in sample size the above-mentioned condition as it is the most stringent. This design ensures to isolate the interaction effect of GLP-1 RA and IPMN while ensuring proper statistical power. Indeed, a-priori power analysis computed with G*Power shows that for our smallest estimation of effect size (i.e., 10-30 patients per condition, 30-60 in total), a 2x2 ANOVA detects a significant medium effect size with a power if 80% and an alpha of 0.05.*

2. No clearly defined compare group.

- We have clarified the study design to explicitly define the comparator groups. The study now follows a 2×2 factorial framework, including four clearly defined groups based on GLP-1 receptor agonist (GLP-1 RA) exposure and IPMN status.
- Page 4-5, lines 148-161: *To assess the impact of GLP-1 RA exposure in patients with IPMNs, we will also include control groups without GLP-1 RA exposure and without IPMNs, resulting in four groups defined by a 2×2 factorial design (GLP-1 RA exposure: yes/no × IPMN: yes/no).*

3. Should consider confounding factors, which is even more important considering the small sample size.

- We acknowledge that the potential impact of confounding factors is particularly relevant given the limited sample size. To address this, groups will be balanced in age, BMI and number of follow-ups.
- Page 7, lines 296-299: *Groups need to balanced in age, BMI, and in their number of follow-ups. If age between groups is unbalanced, as defined by a Cohen’s d > 0.4, outliers will be removed until this threshold is reached. For the number of follow-ups, follow-ups above the limiting factor will be ignored from the data collection to balance the groups if needed.*

4. Elaborate the definition and timing of IPMN progression.

- IPMN progression will be defined as the change of at least one criteria in the Kyoto 2023 Consensus criteria.
- Page 7, lines 305-307: *Timing of progression: T0 will be defined as the first available follow-up examination for patients with IPMN who were not treated with GLP-1 RAs, and as the first available follow-up examination after initiation of GLP-1 RA therapy for patients with IPMN who received GLP-1 RAs.*

**Reviewer #2**:

This study addresses an important and understudied clinical question and may provide valuable preliminary insights into the safety of GLP-1 RAs in patients with IPMNs.

However, substantial methodological limitations, particularly the lack of a control group, necessitate cautious interpretation of the findings. moreover, in my opinion a multicentric and more extensive approach should ave been advisable. With clearer methodological descriptions and a more explicit framing of the study as hypothesis-generating, the manuscript could make a meaningful contribution to the literature and serve as a foundation for future prospective studies.

- In response to the concerns raised, we would like to clarify that a control group has now been added to the study design, resulting in a 2×2 comparative framework that allows for more robust between-group analyses. In addition, the study is multicentric, involving three tertiary care centres, which was specifically implemented to enhance patient recruitment and increase the overall sample size.

**Reviewer #3**: The paper reports the protocol of an observational study on the association of GLP-1 receptor agonist treatment and outcomes of IPMNs. The issue is potentially very interesting and certainly worth investigating. The design of an observational study is the only plausible method for such investigation (a randomized trial would be obviously preferrable, but its development would be unrealistic).

The main issue with this manuscript is that the design of study is not completely clear. Reading the paper, I had the impression that it was a cohort uncontrolled study, aimed at providing descriptive data on the outcomes in patients with IPMN treated with GLP-1 receptor agonists. Then, I found the following sentence:

“To assess the association between GLP-1 RA use and outcomes such as radiological progression or development of acute pancreatitis, we will employ multivariable logistic regression models, adjusting for potential confounders such as age, BMI, diabetes status, and other comorbidities.”

This sentence suggests that patients with IPMN not exposed to GLP-1 RA will also be studied (otherwise, the association between GLP-1 RA and outcomes cannot be assessed). If this is the case (which would make the study much stronger), we definitely need greater detail on this control group (enrolment, etc.).

If patients with IPMN not exposed to GLP-1 RA are used as controls, I recommend to use two different analytical approaches, to obtain a greater reliability of results despite a small sample size:

A. Propensity score-matched cohorts: compare the cohort of GLP-1 RA users to a pronsity-score matched (1:1, 1:2 or even 1:3) of non- GLP-1 RA users with IPMN;

B. Case-control study: identify cases of pancreatitis and progression of IPMN in the whole cohort (GLP-1 RA users and non-users), selected controls (pairwise matched, 1:1, 1:2, 1:3 or higher) matched for main risk factors for either pancreatitis or progression of disease, compare exposure to GLP-1 RA (any exposure; time of exposure) between cases and controls.

- We have substantially revised the study protocol to clarify the study design. Specifically, we have clearly defined the inclusion of a control group consisting of patients with IPMNs who were not exposed to GLP-1 receptor agonists, allowing direct comparison with GLP-1 RA users. We have also expanded and refined the statistical analysis section.

Minor points:

1) In Introduction, some greater background on the possible relationships between GLP-1 receptor agonist treatment and pancreatic cancer should be provided. A recent good review can be found in doi: 10.1038/s41571-026-01135-0.

- We have added the cited review to the Introduction, along with additional recent references, in order to provide a more comprehensive overview of the potential relationship between GLP-1 receptor agonist use and pancreatic cancer.
- Page 3, lines 96-105: *The relationship between GLP-1 RAs and pancreatic cancer remains uncertain. While recent meta-analyses, have not demonstrated a definitive association between GLP-1 RAs use and pancreatic cancer, pharmacovigilance data have suggested a potential signal, with an increased reporting rate of pancreatic malignant neoplasms in association with GLP-1 RAs exposure. The definitive mechanism underlying a potential association between GLP-1 RAs and pancreatic neoplasia has not been established. Proposed hypotheses include a possible link mediated through pancreatitis, a known risk factor for pancreatic cancer, as well as experimental data suggesting potential pro-inflammatory and proliferative effects within pancreatic tissue. However, these findings are inconsistent across studies, and no causal mechanism has been confirmed.*

2) In the Discussion, the authors should elaborate on factors which limit the possibility of enrolling large samples – in particular, warnings on the risk of pancreatitis and pancreatic cancer can porevent the prescription of GLP-1 RA in patients with IPMN.

- We have expanded the Discussion to address factors limiting the inclusion of larger patient cohorts. In particular, we acknowledge that safety concerns and existing warnings regarding pancreatitis and the potential risk of pancreatic malignancy may lead to reluctance in prescribing GLP-1 receptor agonists in patients with known IPMN.
- Page 7, lines 310-380: *Patient identification may be incomplete, as GLP-1 RAs prescriptions may have been initiated by healthcare providers outside the participating institutions or inconsistently documented and therefore not fully captured in institutional records. Furthermore, initiation of the treatment may have been influenced by clinical judgement and recent literature implying a possible link between GLP1 Ras use and pancreatitis or pancreatic cancer, introducing potential prescription bias.*

3) In Discussion, potential limitations of the study should be more clearly stated. Among limitations, limited persistance in therapy with GLP-1 RA could prevent the observation of associations of longer-term treatment with clinical outcomes.

- We have revised the Discussion to more clearly and explicitly state the study limitations. In particular, we now acknowledge that limited persistence with GLP-1 receptor agonist therapy may reduce cumulative exposure and therefore limit the ability to detect potential associations between longer-term treatment and clinical outcomes.
- Page 8, lines 380-383: *In addition, the relatively recent and rapidly increasing widespread use of GLP-1 receptor agonists may limit the ability to assess long-term effects on IPMN evolution. As a result, potential late modifications or clinically relevant changes may not yet be detectable within the available follow-up period.*
