# Supplementary material for: Impact of GLP-1 receptor agonists on patients with intraductal papillary mucinous neoplasms: Study protocol for a multicentric cohort study
Source: PLoS One. 2026 Sep 9;21(9):e0357577. doi: 10.1371/journal.pone.0357577 (PMC13557380; doi:10.1371/journal.pone.0357577)
Supplement: Supplementary file 5 [file pone.0357577.s003.doc]

**Supplementary material 3** SPIRIT schedule of enrollment

|  | **STUDY PERIOD** | | | | | | | |
| --- | --- | --- | --- | --- | --- | --- | --- | --- |
|  | **Enrolment** | **Allocation** | **Post-allocation** | | | | | **Close-out** |
| **TIMEPOINT** | ***Aug-sept 2026*** | **0** | ***Oct-dec 2026*** | ***Nov-jan 2027*** | ***Feb-mar 2027*** | ***Apr-May 2027*** | ***Jul-nov 2027*** | ***December 2027*** |
| **ENROLMENT:** |  |  |  |  |  |  |  |  |
| **Eligibility screen** | X |  |  |  |  |  |  |  |
| **Informed consent check** | X |  |  |  |  |  |  |  |
| **Allocation**  Not applicable |  | - |  |  |  |  |  |  |
| **DATA COLLECTION:** |  |  |  |  |  |  |  |  |
| ***RED Cap*** |  |  | X |  |  |  |  |  |
| ***Transfer radiological records*** |  |  | X |  |  |  |  |  |
| ***Collect endoscopic and histological records*** |  |  | X |  |  |  |  |  |
| **ASSESSMENTS:** |  |  |  |  |  |  |  |  |
| ***Data extraction*** |  |  |  | X |  |  |  |  |
| ***Radiological evaluation*** |  |  |  | X |  |  |  |  |
| ***Statistical analysis*** |  |  |  |  | X |  |  |  |
| ***Manuscript drafting*** |  |  |  |  |  | X |  |  |
| ***Submission/***  ***publication*** |  |  |  |  |  |  | X | X |
