# Supplementary material for: Impact of GLP-1 receptor agonists on patients with intraductal papillary mucinous neoplasms: Study protocol for a multicentric cohort study
Source: PLoS One. 2026 Sep 9;21(9):e0357577. doi: 10.1371/journal.pone.0357577 (PMC13557380; doi:10.1371/journal.pone.0357577)
Supplement: Peer review history

# Peer review: Decision Letter 0

**DOI:** 10.1371/journal.pone.0357577.r001

**Authors:** Giovanni Di Pasquale

15 Apr 2026

Dear Dr. Lagger,

Thank you for submitting your manuscript to PLOS ONE. After careful consideration, we feel that it has merit but does not fully meet PLOS ONE’s publication criteria as it currently stands. Therefore, we invite you to submit a revised version of the manuscript that addresses the points raised during the review process.

Please submit your revised manuscript by May 30 2026 11:59PM. If you will need more time than this to complete your revisions, please reply to this message or contact the journal office at plosone@plos.org. When you're ready to submit your revision, log on to https://www.editorialmanager.com/pone/ and select the 'Submissions Needing Revision' folder to locate your manuscript file.

- A letter that responds to each point raised by the academic editor and reviewer(s). You should upload this letter as a separate file labeled 'Response to Reviewers'.
- A marked-up copy of your manuscript that highlights changes made to the original version. You should upload this as a separate file labeled 'Revised Manuscript with Track Changes'.
- An unmarked version of your revised paper without tracked changes. You should upload this as a separate file labeled 'Manuscript'.

If you would like to make changes to your financial disclosure, please include your updated statement in your cover letter. Guidelines for resubmitting your figure files are available below the reviewer comments at the end of this letter.

If applicable, we recommend that you deposit your laboratory protocols in protocols.io to enhance the reproducibility of your results. Protocols.io assigns your protocol its own identifier (DOI) so that it can be cited independently in the future. For instructions see: https://journals.plos.org/plosone/s/submission-guidelines#loc-laboratory-protocols. Additionally, PLOS ONE offers an option for publishing peer-reviewed Lab Protocol articles, which describe protocols hosted on protocols.io. Read more information on sharing protocols at https://plos.org/protocols?utm_medium=editorial-email&utm_source=authorletters&utm_campaign=protocols.

As the corresponding author, your ORCID iD is verified in the submission system and will appear in the published article. PLOS supports the use of ORCID, and we encourage all coauthors to register for an ORCID iD and use it as well. Please encourage your coauthors to verify their ORCID iD within the submission system before final acceptance, as unverified ORCID iDs will not appear in the published article. *Only*  the individual author can complete the verification step; PLOS staff *cannot*  verify ORCID iDs on behalf of authors.

We look forward to receiving your revised manuscript.

Kind regards,

Giovanni Di Pasquale

Academic Editor

PLOS One

Journal Requirements:

When submitting your revision, we need you to address these additional requirements.

1. Please ensure that your manuscript meets PLOS ONE's style requirements, including those for file naming. The PLOS ONE style templates can be found at

https://journals.plos.org/plosone/s/file?id=wjVg/PLOSOne_formatting_sample_main_body.pdf and https://journals.plos.org/plosone/s/file?id=ba62/PLOSOne_formatting_sample_title_authors_affiliations.pdf

2. Your ethics statement should only appear in the Methods section of your manuscript. If your ethics statement is written in any section besides the Methods, please delete it from any other section.

3. Please provide a complete Data Availability Statement in the submission form, ensuring you include all necessary access information or a reason for why you are unable to make your data freely accessible. If your research concerns only data provided within your submission, please write "All data are in the manuscript and/or supporting information files" as your Data Availability Statement.

4. We note that Figure 1 in your submission contain copyrighted images. All PLOS content is published under the Creative Commons Attribution License (CC BY 4.0), which means that the manuscript, images, and Supporting Information files will be freely available online, and any third party is permitted to access, download, copy, distribute, and use these materials in any way, even commercially, with proper attribution. For more information, see our copyright guidelines: http://journals.plos.org/plosone/s/licenses-and-copyright.

We require you to either (1) present written permission from the copyright holder to publish these figures specifically under the CC BY 4.0 license, or (2) remove the figures from your submission:

a. You may seek permission from the original copyright holder of Figure 1 to publish the content specifically under the CC BY 4.0 license.

We recommend that you contact the original copyright holder with the Content Permission Form (http://journals.plos.org/plosone/s/file?id=7c09/content-permission-form.pdf) and the following text:

“I request permission for the open-access journal PLOS ONE to publish XXX under the Creative Commons Attribution License (CCAL) CC BY 4.0 (http://creativecommons.org/licenses/by/4.0/). Please be aware that this license allows unrestricted use and distribution, even commercially, by third parties. Please reply and provide explicit written permission to publish XXX under a CC BY license and complete the attached form.”

Please upload the completed Content Permission Form or other proof of granted permissions as an "Other" file with your submission.

In the figure caption of the copyrighted figure, please include the following text: “Reprinted from [ref] under a CC BY license, with permission from [name of publisher], original copyright [original copyright year].”

b. If you are unable to obtain permission from the original copyright holder to publish these figures under the CC BY 4.0 license or if the copyright holder’s requirements are incompatible with the CC BY 4.0 license, please either i) remove the figure or ii) supply a replacement figure that complies with the CC BY 4.0 license. Please check copyright information on all replacement figures and update the figure caption with source information. If applicable, please specify in the figure caption text when a figure is similar but not identical to the original image and is therefore for illustrative purposes only.

5. Please include a caption for figure 1.

6. Please include captions for your Supporting Information files at the end of your manuscript, and update any in-text citations to match accordingly. Please see our Supporting Information guidelines for more information: http://journals.plos.org/plosone/s/supporting-information.

7. If the reviewer comments include a recommendation to cite specific previously published works, please review and evaluate these publications to determine whether they are relevant and should be cited. There is no requirement to cite these works unless the editor has indicated otherwise.

[Note: HTML markup is below. Please do not edit.]

Reviewers' comments:

Reviewer's Responses to Questions

**Comments to the Author**

1. Does the manuscript provide a valid rationale for the proposed study, with clearly identified and justified research questions?

Reviewer #1: Partly

Reviewer #2: Yes

Reviewer #3: Yes

**********

2. Is the protocol technically sound and planned in a manner that will lead to a meaningful outcome and allow testing the stated hypotheses?

Reviewer #1: Partly

Reviewer #2: Yes

Reviewer #3: Partly

**********

3. Is the methodology feasible and described in sufficient detail to allow the work to be replicable?

Reviewer #1: No

Reviewer #2: Yes

Reviewer #3: Yes

**********

4. Have the authors described where all data underlying the findings will be made available when the study is complete?

The PLOS Data policy

Reviewer #1: No

Reviewer #2: Yes

Reviewer #3: No

**********

5. Is the manuscript presented in an intelligible fashion and written in standard English?

Reviewer #1: Yes

Reviewer #2: Yes

Reviewer #3: Yes

**********

Please use the space provided to explain your answers to the questions above and, if applicable, provide comments about issues authors must address before this protocol can be accepted for publication. You may also include additional comments for the author, including concerns about research or publication ethics.

You may also provide optional suggestions and comments to authors that they might find helpful in planning their study.

Reviewer #1: In the paper, Melissa et al presents a multicentric retrospective cohort study protocol. They aim to study the association between the usage of GLP-1 receptor agonist (GLP-1 RA) and the progression of intraductal papillary mucinous neoplasms (IPMNs). The topic is clinically relevant given the increasing prevalence of IPMNs and the widespread use of GLP-1 RAs for diabetes and obesity. The protocol is generally well written, follows SPIRIT 2013 guidelines, and clearly describes objectives, inclusion criteria, outcomes, and planned analyses. However, there are important methodological and interpretative limitations, particularly related to sample size, comparator definition, confounding control, and feasibility of the planned statistical analyses, that should be addressed or clarified to strengthen the protocol and avoid overinterpretation of results.

1. Expected sample size is too limited.

2. No clearly defined compare group.

3. Should consider confounding factors, which is even more important considering the small sample size.

4. Elaborate the definition and timing of IPMN progression.

Reviewer #2: this study addresses an important and understudied clinical question and may provide valuable preliminary insights into the safety of GLP-1 RAs in patients with IPMNs.

However, substantial methodological limitations, particularly the lack of a control group, necessitate cautious interpretation of the findings. moreover, in my opinion a multicentric and more extensive approach should ave been advisable.

With clearer methodological descriptions and a more explicit framing of the study as hypothesis-generating, the manuscript could make a meaningful contribution to the literature and serve as a foundation for future prospective studies.

Reviewer #3: The paper reports the protocol of an observational study on the association of GLP-1 receptor agonist treatment and outcomes of IPMNs. The issue is potentially very interesting and certainly worth investigating. The design of an observational study is the only plausible method for such investigation (a randomized trial would be obviously preferrable, but its development would be unrealistic).

The main issue with this manuscript is that the design of study is not completely clear. Reading the paper, I had the impression that it was a cohort uncontrolled study, aimed at providing descriptive data on the outcomes in patients with IPMN treated with GLP-1 receptor agonists. Then, I found the following sentence:

“To assess the association between GLP-1 RA use and outcomes such as radiological progression or development of acute pancreatitis, we will employ multivariable logistic regression models, adjusting for potential confounders such as age, BMI, diabetes status, and other comorbidities.”

This sentence suggests that patients with IPMN not exposed to GLP-1 RA will also be studied (otherwise, the association between GLP-1 RA and outcomes cannot be assessed). If this is the case (which would make the study much stronger), we definitely need greater detail on this control group (enrolment, etc.).

If patients with IPMN not exposed to GLP-1 RA are used as controls, I recommend to use two different analytical approaches, to obtain a greater reliability of results despite a small sample size:

A. Propensity score-matched cohorts: compare the cohort of GLP-1 RA users to a pronsity-score matched (1:1, 1:2 or even 1:3) of non- GLP-1 RA users with IPMN;

B. Case-control study: identify cases of pancreatitis and progression of IPMN in the whole cohort (GLP-1 RA users and non-users), selected controls (pairwise matched, 1:1, 1:2, 1:3 or higher) matched for main risk factors for either pancreatitis or progression of disease, compare exposure to GLP-1 RA (any exposure; time of exposure) between cases and controls.

Minor points:

1) In Introduction, some greater background on the possible relationships between GLP-1 receptor agonist treatment and pancreatic cancer should be provided. A recent good review can be found in doi: 10.1038/s41571-026-01135-0.

2) In the Discussion, the authors should elaborate on factors which limit the possibility of enrolling large samples – in particular, warnings on the risk of pancreatitis and pancreatic cancer can porevent the prescription of GLP-1 RA in patients with IPMN.

3) In Discussion, potential limitations of the study should be more clearly stated. Among limitations, limited persistance in therapy with GLP-1 RA could prevent the observation of associations of longer-term treatment with clinical outcomes.

**********

what does this mean?). If published, this will include your full peer review and any attached files.

If you choose “no”, your identity will remain anonymous but your review may still be made public.

**Do you want your identity to be public for this peer review?**  For information about this choice, including consent withdrawal, please see our Privacy Policy

Reviewer #1: No

Reviewer #2: No

Reviewer #3: No

**********

[NOTE: If reviewer comments were submitted as an attachment file, they will be attached to this email and accessible via the submission site. Please log into your account, locate the manuscript record, and check for the action link "View Attachments". If this link does not appear, there are no attachment files.]

To ensure your figures meet our technical requirements, please review our figure guidelines: https://journals.plos.org/plosone/s/figures

You may also use PLOS’s free figure tool, NAAS, to help you prepare publication quality figures: https://journals.plos.org/plosone/s/figures#loc-tools-for-figure-preparation.

NAAS will assess whether your figures meet our technical requirements by comparing each figure against our figure specifications.

# Author comment: Author response to Decision Letter 1

**DOI:** 10.1371/journal.pone.0357577.r002

26 Jul 2026

Reviewer #1:

In the paper, Melissa et al presents a multicentric retrospective cohort study protocol. They aim to study the association between the usage of GLP-1 receptor agonist (GLP-1 RA) and the progression of intraductal papillary mucinous neoplasms (IPMNs). The topic is clinically relevant given the increasing prevalence of IPMNs and the widespread use of GLP-1 RAs for diabetes and obesity. The protocol is generally well written, follows SPIRIT 2013 guidelines, and clearly describes objectives, inclusion criteria, outcomes, and planned analyses. However, there are important methodological and interpretative limitations, particularly related to sample size, comparator definition, confounding control, and feasibility of the planned statistical analyses, that should be addressed or clarified to strengthen the protocol and avoid overinterpretation of results.

1. Expected sample size is too limited.

� Following the introduction of control groups, the expected total sample size has been increased ranging between approximately 30 and 60 patients. We acknowledge that this sample size is constrained by the rarity of concomitant GLP-1 receptor agonist exposure and IPMN diagnosis. However, the multicentric design of this retrospective study, involving three tertiary care centres, was specifically implemented to maximise patient identification and recruitment. Despite these efforts, we recognise that the study remains exploratory in nature and that the limited sample size represents an inherent limitation.

� Page 6-7, lines 243-281: The sample size is expected to be between 10 and 20 patients for the condition with GLP-1 RAs and IPMN, due to the rare nature of the patient population with both IPMN and GLP-1 RA use. The other conditions (GLP-1 RA & no-IPMN, no GLP-1 RA & IPMN) will match in sample size the above-mentioned condition as it is the most stringent. This design ensures to isolate the interaction effect of GLP-1 RA and IPMN while ensuring proper statistical power. Indeed, a-priori power analysis computed with G*Power shows that for our smallest estimation of effect size (i.e., 10-30 patients per condition, 30-60 in total), a 2x2 ANOVA detects a significant medium effect size with a power if 80% and an alpha of 0.05.

2. No clearly defined compare group.

� We have clarified the study design to explicitly define the comparator groups. The study now follows a 2×2 factorial framework, including four clearly defined groups based on GLP-1 receptor agonist (GLP-1 RA) exposure and IPMN status.

� Page 4-5, lines 148-161: To assess the impact of GLP-1 RA exposure in patients with IPMNs, we will also include control groups without GLP-1 RA exposure and without IPMNs, resulting in four groups defined by a 2×2 factorial design (GLP-1 RA exposure: yes/no × IPMN: yes/no).

3. Should consider confounding factors, which is even more important considering the small sample size.

� We acknowledge that the potential impact of confounding factors is particularly relevant given the limited sample size. To address this, groups will be balanced in age, BMI and number of follow-ups.

� Page 7, lines 296-299: Groups need to balanced in age, BMI, and in their number of follow-ups. If age between groups is unbalanced, as defined by a Cohen’s d > 0.4, outliers will be removed until this threshold is reached. For the number of follow-ups, follow-ups above the limiting factor will be ignored from the data collection to balance the groups if needed.

4. Elaborate the definition and timing of IPMN progression.

� IPMN progression will be defined as the change of at least one criteria in the Kyoto 2023 Consensus criteria.

� Page 7, lines 305-307: Timing of progression: T0 will be defined as the first available follow-up examination for patients with IPMN who were not treated with GLP-1 RAs, and as the first available follow-up examination after initiation of GLP-1 RA therapy for patients with IPMN who received GLP-1 RAs.

Reviewer #2:

This study addresses an important and understudied clinical question and may provide valuable preliminary insights into the safety of GLP-1 RAs in patients with IPMNs.

However, substantial methodological limitations, particularly the lack of a control group, necessitate cautious interpretation of the findings. moreover, in my opinion a multicentric and more extensive approach should ave been advisable. With clearer methodological descriptions and a more explicit framing of the study as hypothesis-generating, the manuscript could make a meaningful contribution to the literature and serve as a foundation for future prospective studies.

� In response to the concerns raised, we would like to clarify that a control group has now been added to the study design, resulting in a 2×2 comparative framework that allows for more robust between-group analyses. In addition, the study is multicentric, involving three tertiary care centres, which was specifically implemented to enhance patient recruitment and increase the overall sample size.

Reviewer #3: The paper reports the protocol of an observational study on the association of GLP-1 receptor agonist treatment and outcomes of IPMNs. The issue is potentially very interesting and certainly worth investigating. The design of an observational study is the only plausible method for such investigation (a randomized trial would be obviously preferrable, but its development would be unrealistic).

The main issue with this manuscript is that the design of study is not completely clear. Reading the paper, I had the impression that it was a cohort uncontrolled study, aimed at providing descriptive data on the outcomes in patients with IPMN treated with GLP-1 receptor agonists. Then, I found the following sentence:

“To assess the association between GLP-1 RA use and outcomes such as radiological progression or development of acute pancreatitis, we will employ multivariable logistic regression models, adjusting for potential confounders such as age, BMI, diabetes status, and other comorbidities.”

This sentence suggests that patients with IPMN not exposed to GLP-1 RA will also be studied (otherwise, the association between GLP-1 RA and outcomes cannot be assessed). If this is the case (which would make the study much stronger), we definitely need greater detail on this control group (enrolment, etc.).

If patients with IPMN not exposed to GLP-1 RA are used as controls, I recommend to use two different analytical approaches, to obtain a greater reliability of results despite a small sample size:

A. Propensity score-matched cohorts: compare the cohort of GLP-1 RA users to a pronsity-score matched (1:1, 1:2 or even 1:3) of non- GLP-1 RA users with IPMN;

B. Case-control study: identify cases of pancreatitis and progression of IPMN in the whole cohort (GLP-1 RA users and non-users), selected controls (pairwise matched, 1:1, 1:2, 1:3 or higher) matched for main risk factors for either pancreatitis or progression of disease, compare exposure to GLP-1 RA (any exposure; time of exposure) between cases and controls.

� We have substantially revised the study protocol to clarify the study design. Specifically, we have clearly defined the inclusion of a control group consisting of patients with IPMNs who were not exposed to GLP-1 receptor agonists, allowing direct comparison with GLP-1 RA users. We have also expanded and refined the statistical analysis section.

Minor points:

1) In Introduction, some greater background on the possible relationships between GLP-1 receptor agonist treatment and pancreatic cancer should be provided. A recent good review can be found in doi: 10.1038/s41571-026-01135-0.

� We have added the cited review to the Introduction, along with additional recent references, in order to provide a more comprehensive overview of the potential relationship between GLP-1 receptor agonist use and pancreatic cancer.

� Page 3, lines 96-105: The relationship between GLP-1 RAs and pancreatic cancer remains uncertain. While recent meta-analyses, have not demonstrated a definitive association between GLP-1 RAs use and pancreatic cancer, pharmacovigilance data have suggested a potential signal, with an increased reporting rate of pancreatic malignant neoplasms in association with GLP-1 RAs exposure. The definitive mechanism underlying a potential association between GLP-1 RAs and pancreatic neoplasia has not been established. Proposed hypotheses include a possible link mediated through pancreatitis, a known risk factor for pancreatic cancer, as well as experimental data suggesting potential pro-inflammatory and proliferative effects within pancreatic tissue. However, these findings are inconsistent across studies, and no causal mechanism has been confirmed.

2) In the Discussion, the authors should elaborate on factors which limit the possibility of enrolling large samples – in particular, warnings on the risk of pancreatitis and pancreatic cancer can porevent the prescription of GLP-1 RA in patients with IPMN.

� We have expanded the Discussion to address factors limiting the inclusion of larger patient cohorts. In particular, we acknowledge that safety concerns and existing warnings regarding pancreatitis and the potential risk of pancreatic malignancy may lead to reluctance in prescribing GLP-1 receptor agonists in patients with known IPMN.

� Page 7, lines 310-380: Patient identification may be incomplete, as GLP-1 RAs prescriptions may have been initiated by healthcare providers outside the participating institutions or inconsistently documented and therefore not fully captured in institutional records. Furthermore, initiation of the treatment may have been influenced by clinical judgement and recent literature implying a possible link between GLP1 Ras use and pancreatitis or pancreatic cancer, introducing potential prescription bias.

3) In Discussion, potential limitations of the study should be more clearly stated. Among limitations, limited persistance in therapy with GLP-1 RA could prevent the observation of associations of longer-term treatment with clinical outcomes.

� We have revised the Discussion to more clearly and explicitly state the study limitations. In particular, we now acknowledge that limited persistence with GLP-1 receptor agonist therapy may reduce cumulative exposure and therefore limit the ability to detect potential associations between longer-term treatment and clinical outcomes.

� Page 8, lines 380-383: In addition, the relatively recent and rapidly increasing widespread use of GLP-1 receptor agonists may limit the ability to assess long-term effects on IPMN evolution. As a result, potential late modifications or clinically relevant changes may not yet be detectable within the available follow-up period.

Attachment Submitted filename: Response Reviewers.docx

# Editor report: Decision Letter 1

**DOI:** 10.1371/journal.pone.0357577.r003

**Authors:** Giovanni Di Pasquale

20 Aug 2026

Impact of GLP-1 Receptor Agonists on Patients with Intraductal Papillary Mucinous Neoplasms: Study Protocol for a Multicentric Cohort Study

PONE-D-25-43564R1

Dear Dottoressa Melissa Lagger

After reading the revised manuscript I am satisfied with the chances and improvements.

We’re pleased to inform you that your manuscript has been judged scientifically suitable for publication and will be formally accepted for publication once it meets all outstanding technical requirements.

Within one week, you’ll receive an e-mail detailing the required amendments. When these have been addressed, you’ll receive a formal acceptance letter and your manuscript will be scheduled for publication.

An invoice will be generated when your article is formally accepted. Please note, if your institution has a publishing partnership with PLOS and your article meets the relevant criteria, all or part of your publication costs will be covered. Please make sure your user information is up-to-date by logging into Editorial Manager at Editorial Manager® and clicking the ‘Update My Information' link at the top of the page. For questions related to billing, please contact billing support.

If your institution or institutions have a press office, please notify them about your upcoming paper to help maximize its impact. If they’ll be preparing press materials, please inform our press team as soon as possible -- no later than 48 hours after receiving the formal acceptance. Your manuscript will remain under strict press embargo until 2 pm Eastern Time on the date of publication. For more information, please contact onepress@plos.org.

Kind regards,

Giovanni Di Pasquale

Academic Editor

PLOS One

Additional Editor Comments (optional):

Reviewers' comments:

# Editor report: Acceptance letter

**DOI:** 10.1371/journal.pone.0357577.r004

**Authors:** Giovanni Di Pasquale

PONE-D-25-43564R1

PLOS One

Dear Dr. Lagger,

I'm pleased to inform you that your manuscript has been deemed suitable for publication in PLOS One. Congratulations! Your manuscript is now being handed over to our production team.

At this stage, our production department will prepare your paper for publication. This includes ensuring the following:

* All references, tables, and figures are properly cited

* All relevant supporting information is included in the manuscript submission,

* There are no issues that prevent the paper from being properly typeset

You will receive further instructions from the production team, including instructions on how to review your proof when it is ready. Please keep in mind that we are working through a large volume of accepted articles, so please give us a few days to review your paper and let you know the next and final steps.

Lastly, if your institution or institutions have a press office, please let them know about your upcoming paper now to help maximize its impact. If they'll be preparing press materials, please inform our press team within the next 48 hours. Your manuscript will remain under strict press embargo until 2 pm Eastern Time on the date of publication. For more information, please contact onepress@plos.org.

You will receive an invoice from PLOS for your publication fee after your manuscript has reached the completed accept phase. If you receive an email requesting payment before acceptance or for any other service, this may be a phishing scheme. Learn how to identify phishing emails and protect your accounts at https://explore.plos.org/phishing.

If we can help with anything else, please email us at customercare@plos.org.

Thank you for submitting your work to PLOS One and supporting open access.

Kind regards,

PLOS One Editorial Office Staff

on behalf of

Dr. Giovanni Di Pasquale

Academic Editor

PLOS One
